# The first mitochondrial genome for the subfamily Podopinae (Hemiptera: Pentatomidae) and its phylogenetic implications

**DOI:** 10.1080/23802359.2017.1310605

**Published:** 2017-04-09

**Authors:** Juan Wang, Li Zhang, Xing-Zhuo Yang, Min-Qiang Zhou, Ming-Long Yuan

**Affiliations:** State Key Laboratory of Grassland Agro-Ecosystems, College of Pastoral Agricultural Science and Technology, Lanzhou University, Lanzhou, Gansu, People's Republic of China

**Keywords:** Heteroptera, Pentatomoidea, *Graphosoma rubrolineata*, mitochondrial DNA, phylogeny

## Abstract

Here, we determined the complete mitogenome of *Graphosoma rubrolineata*, as the first representative for the subfamily Podopinae. This mitogenome is 15,633 bp long and contains 37 typical mitochondrial genes. The genome size, gene arrangement, A + T content, codon usage and secondary structures of 22 tRNAs of the *G. rubrolineata* mitogenome were similar to that of other sequenced pentatomoids. This mitogenome exhibited a reverse nucleotide strand bias, i.e. positive GC-skew (0.021) and negative AT-skew (−0.086). Phylogenetic analyses based on mitogenomic data strongly supported the monophyly of each of the five superfamilies within Pentatomomorpha and recognized a phylogeny of (Aradoidea + (Pentatomoidea + (Lygaeoidea + (Pyrrhocoroidea + Coreoidea)))). However, *G. rubrolineata* clustered with three Pentatominae species, suggesting that Pentatominae probably was not monophyletic, or Podopinae may not be a valid taxonomic group. The mitogenome sequence of *G. rubrolineata* could contribute for better understanding of population genetics and evolution of this insect pest.

Pentatomidae is one of the most diverse groups in Heteroptera and of great economic importance (Rider 2011). To date, all the five pentatomid mitogenomes are from Pentatominae, which limits our understanding on the diversity and phylogeny of Pentatomidae. Here, we determined the complete mitogenome of *Graphosoma rubrolineata*, an important pest on vegetables and medicinal herbs in China. Adult specimens of *G. rubrolineata* were collected from Qingyang City, Gansu Province, China, in July 2015. Samples have been deposited in the State Key Laboratory of Grassland Agro-Ecosystems, College of Pastoral Agricultural Science and Technology, Lanzhou University, Lanzhou, China.

This mitogenome was a typical circular DNA molecule with 15,633 bp in length (GenBank accession no. KX267740) and contained 37 typical mitochondrial genes: 13 protein-coding genes (PCGs), 22 transfer RNA genes (tRNAs), the large and small ribosomal RNA unit genes (*rrnL* and *rrnS*). All genes were arranged in the same order as the inferred insect ancestral mitogenome (Boore 1999). The nucleotide composition of the *G. rubrolineata* mitogenome was significantly biased towards A and T, with a 74.98% A + T content in average. This mitogenome presented a negative AT-skew (−0.086) and a positive GC-skew (0.021), which was contrary to that of most insect mitogenomes. 10 PCGs started with a typical ATN codon and the remaining three PCGs (*cox1*, *atp8*, and *nad1*) began with TTG. Eight PCGs terminated with TAA, four terminated with an incomplete stop codon T––, whereas *nad4* terminated with TAG, another frequently used stop codon in insect mitogenomes.

All of the 22 tRNAs had a typical cloverleaf structure, except for *trnS1* (AGN) in which its dihydrouridine arm simply formed a loop. In addition, *trnS1* in the *G. rubrolineata* mitogenome presented an unusual anticodon stem (9 bp vs. the normal 5 bp) and a bulged nucleotide in the middle of the AC stem, which was a common feature in true bugs (Zhang et al. 2013; Yuan et al. 2015a, 2015b). The *rrnL* was 1277 bp long with an A + T content of 78.8%, and the *rrnS* was 796 bp with an A + T content of 77.5%.

**Figure 1.**
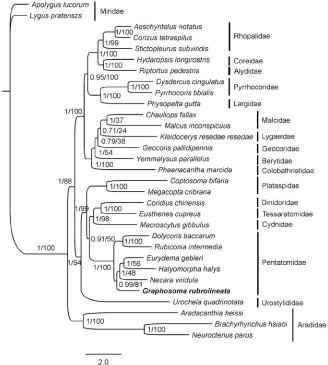
Mitochondrial phylogeny of 29 Pentatomomorpha species based on the concatenated nucleotide sequences of 13 mitochondrial protein-coding genes. Bayesian inference and maximum-likelihood analyses recover the same three topology. Numbers on branches are Bayesian posterior probabilities (left) and bootstrap values (right).

Phylogenetic analyses of 29 Pentatomomorpha species with Bayesian inference and maximum-likelihood methods using the concatenated nucleotide sequences of 13 mitochondrial PCGs resulted in identical tree topology (Figure 1). The monophyly of each of the five superfamilies was highly supported and a phylogeny of (Aradoidea + (Pentatomoidea + (Lygaeoidea + (Coreoidea + Pyrrhocoroidea)))) was recovered, which was consistent with our previous studies (Yuan et al. 2015a, 2015b). Each of the six families within Pentatomoidae was recovered as monophyletic group and Pentatomidae was more closely related to Cydnidae. Although *G. rubrolineata* was from the subfamily Podopinae, this species mixed with species from the subfamily Pentatominae. This suggested that Pentatominae might not be monophyletic, or Podopinae may not be an effective taxonomic category. Therefore, further studies are needed to sequence more species from Podopinae and other subfamilies, which will enhance our understanding of molecular phylogeny in Pentatomidae. This is the first sequenced mitogenome from the subfamily Podopinae and the mitogenomic data of *G. rubrolineata* could contribute for better understanding of population genetics and evolution of this insect pest.
